# P31. Genomic profiling and functional characterisation of a new myeloid cell type enriched in renal cell carcinoma

**DOI:** 10.1186/2051-1426-2-S2-P22

**Published:** 2014-03-12

**Authors:** D Brech, M Irmler, T Straub, P Bruns, C Jaekel, E Noessner

**Affiliations:** 1 Helmholtz Zentrum Muenchen, Institute of Molecular Immunology, Munich, Germany; 2 Helmholtz Zentrum Muenchen, Institute of Experimental Genetics, Munich, Germany; 3 Ludwig-Maximilians-University, Adolf Budenandt Institute, Munich, Germany; 4 Helmholtz Zentrum Muenchen, Institute of Bioinformatics and Systems Biology, Munich, Germany; 5 Ludwig-Maximilians-University, Clinical Biochemistry, Munich, Germany

## 

Dendritic cells (DCs) are central players of the immune response because they regulate both innate and adaptive immune cells. As part of the leukocyte infiltrate of various tumours they can initiate antitumour immune responses through the activation of tumour-specific T cells.

However, tumours employ strategies to evade the immune response by manipulating the differentiation and activation of DCs.

Previously we described 'enriched-in-renal-carcinoma DCs' (ercDCs) as an unusual CD14^+^CD209^+^ expressing myeloid cell type that displays characteristics of both DCs and macrophages. As one example, ercDCs showed good antigen cross presentation which is a prototypic DC function and a prerequisite for the activation of T effector cells in local tumour tissue. To better understand the relationship of ercDCs to other myeloid cell types and to get insight in functional pathways operative in ercDCs we performed transcriptome analysis using Affymetrix® gene arrays comparing ercDCs, cDCs, proinflammatory M1 and GM-CSF macrophages and alternatively activated M2 macrophages. Cell types were generated in parallel from monocytes of 15 healthy donors. RNA was extracted using the Qiagen® RNeasy Micro kit. To reduce population variability, RNA from each cell type- from 5 donors- was pooled and 3 biological replicates generated. Clustering of normalised values showed that ercDCs differ from the other myeloid cell types. Further analysis included heat mapping and creation of signaling pathways using pathvisio software. Focusing on the cross presentation pathway, a gene list was assembled through literature mining and each gene was assigned an expression value using microarray data. A comparison using the heat map representation revealed that the expression pattern of ercDCs was most similar to GM-CSF macrophages and least to M1 macrophages. Comparison based on fold changes generated by normalisation to cDCs revealed highest similarity between ercDCs and M2 macrophages. Particularly, transcription of genes involved in receptor-mediated endocytosis and the vacuolar pathway were upregulated in ercDCs indicative that cross presentation could be active in ercDCs.

